# Evaluation of a management platform for extended care of type 2 diabetes

**DOI:** 10.1186/s13098-025-01835-0

**Published:** 2025-07-02

**Authors:** Qing Zhou, Li Zou, Yan Gao, Guo-Hong Zhu

**Affiliations:** 1 Department of Endocrinology, Taizhou People’s Hospital, Taizhou, 225300 Jiangsu China; 2 Department of Nephrology, Taizhou People’s Hospital, No. 366, Taihu Road, Hailing District, Taizhou, 225300 Jiangsu China

**Keywords:** Diabetes, Diabetes chronic disease management platform, Extended care, Blood glucose, Self-management ability

## Abstract

**Objective:**

This study aims to evaluate the efficacy of a diabetes chronic disease management platform in extended care for patients with diabetes.

**Methods:**

A cohort of 150 patients with type 2 diabetes (mean age, 51 years) were selected and randomly assigned to an observation group and a control group, each comprising 75 participants, from September 2023 to September 2024. The control group received standard extended care, while the observation group received extended care integrated with the diabetes chronic disease management platform. The two groups were compared in terms of blood glucose levels, emotional state, self-management ability, and quality of life at discharge and three months after the intervention. Additionally, the incidence of hypoglycemia and hospital readmission within three months after discharge were recorded and analyzed.

**Results:**

Three months after the intervention, both groups exhibited reductions in fasting blood glucose, 2-hour postprandial blood glucose, glycated hemoglobin, and scores on the Self-Rating Anxiety Scale and Self-Rating Depression Scale compared to discharge levels. However, the observation group had significantly lower values than the control group (*p* < 0.05). Scores on the Summary of Diabetes Self-Care Activities decreased in both groups compared to discharge levels, but the observation group had relatively higher scores (*p* < 0.05). Scores on the General Quality of Life Inventory-74 increased in both groups compared to discharge, with the observation group demonstrating significantly higher scores across all dimensions (*p* < 0.05). Additionally, the incidence rates of hypoglycemia and hospital readmission were lower in the observation group than in the control group (*p* < 0.05).

**Conclusion:**

The diabetes chronic disease management platform improves the effectiveness of extended care interventions in managing blood glucose levels, emotional state, self-management ability, and quality of life in patients with diabetes, while also reducing the risk of hypoglycemia and hospital readmission after discharge.

## Introduction

Diabetes is a prevalent chronic condition characterized by elevated blood glucose levels and can be classified into different types based on specific causes and pathogenic mechanisms. Among these, type 2 diabetes is the most common form [1]. According to relevant surveys, the incidence of diabetes in China has been increasing in recent years [2]. Prolonged hyperglycemia can adversely affect multiple organ systems, including the heart, brain, and kidneys, leading to various complications that significantly impact a patient’s quality of life. Additionally, studies indicate that the prevalence of diabetes is shifting toward younger populations, with a rising proportion of middle-aged and young patients [3]. Consequently, diabetes and its associated complications pose a major public health challenge in China. Patients with diabetes often have limited awareness of disease management and are influenced by various factors such as economic constraints and lifestyle habits, making it difficult for them to adhere to medical recommendations regarding diet, exercise, and medication management after discharge, thereby resulting in suboptimal disease control [4]. 

Extended care is an advanced nursing model that extends patient care from hospitalization to the post-discharge period. Its goal is to provide patients with comprehensive nursing interventions, facilitating better rehabilitation outcomes [5]. In recent years, extended care has been increasingly utilized in diabetes management to regulate blood glucose levels. However, traditional extended care methods remain limited, often relying on passive patient participation and lacking diverse approaches [6]. With the continuous advancement of internet-based medical care, online communication between healthcare providers and patients is becoming more common. Medical staff can use online platforms to understand and monitor changes in patients’ conditions. Patients can also receive professional medical treatment and intervention in a “face-to-face” manner at home through these platforms, which is conducive to improving patients’ self-management skills and disease control outcomes. Previously, patients could only passively accept extended care, and the communication between medical staff and patients was limited to a single form. It mainly relied on phone follow-ups, discharge guidance, and outpatient re-examinations, with communication being dominated by the one-sided oral instructions from medical staff. After the application of the diabetes management platform, patients have a greater sense of participation and a more comprehensive understanding of their disease control. Digital platforms for chronic disease management are emerging as a promising strategy for controlling disease progression [7]. Based on this, the present study utilizes a diabetes chronic disease management platform to assess extended care for patients with diabetes. The objective is to evaluate the platform’s clinical effectiveness and provide additional evidence for clinical practice.

## Materials and methods

### General information

Using the formula for comparing the means of two independent samples, with the research significance level set at 0.05 and the confidence level at 90%, the required sample size was calculated to be 125 cases. Considering a 20% loss rate, the sample size was increased accordingly, and the final sample size was set at 150 cases. A cohort of 150 patients with diabetes who received treatment at the hospital from September 2023 to September 2024 were selected. The inclusion criteria were as follows: (1) meeting the relevant diagnostic criteria for type 2 diabetes [8]; (2) having normal consciousness and cognitive function, with the ability to comprehend and communicate effectively; (3) aged between 18 and 75 years; (4) possessing basic proficiency in using internet platforms such as WeChat; and (5) being familiar with the research content and voluntarily signing an informed consent form. The exclusion criteria were: (1) presence of multiple acute complications; (2) restricted physical activity; (3) coexistence of malignant tumors; (4) inability to operate online platforms; and (5) pregnancy or lactation. A table consisting of 150 patients meeting the criteria was generated based on randomly generated numbers. The starting position of the number table was the 3rd column of the 5th row. Starting from this position, read the numbers in the order from left to right. Odd numbers were assigned to the experimental group, and even numbers to the control group. The enrolled patients were randomly assigned to either the observation group or the control group, with 75 patients in each. There was no statistically significant difference in baseline data between the observation and control groups (*p* > 0.05), as presented in Table 1.

**Table 1:** Comparison of general information between the two groups

Group	Number of cases	Sex		Age	Body Mass Index (BMI)	Education level			Duration of disease (years)
		Male	Female			Junior high school and below	Technical secondary school and high school	College and above	
Observation group	75	43(57.33)	32(42.67)	51.19 ± 5.71	23.39 ± 1.16	7(9.33)	21(28.00)	47(62.67)	8.31 ± 2.37
Control group	75	45(60.00)	30(40.00)	50.93 ± 6.12	23.45 ± 1.08	6(8.00)	26(34.67)	43(57.33)	8.11 ± 2.42
χ2/t		0.110		0.269	0.343	0.787			0.511
P		0.740		0.788	0.732	0.675			0.610

### Methods

#### Control group

Patients received traditional extended care interventions, which involved providing a health manual and discharge education on the day of discharge. They were informed about medication-related precautions and the significance of blood glucose monitoring. After discharge, regular telephone follow-ups and outpatient reviews were conducted to assess patients’ adherence to dietary guidelines, medication use, physical activity, and blood glucose management while offering appropriate guidance.

#### Observation group

Patients received extended care through the diabetes chronic disease management platform, with the specific intervention process outlined as follows:

(1) A diabetes management platform was developed using the WeChat official account, incorporating the following sections: patient personal profiles, blood glucose monitoring, online follow-up appointment scheduling, extended care service updates, interactive communication, health columns, and real-time online guidance.

(2) An extended care service team was established, consisting of a guidance team and a working team. The guidance team primarily included experts from the endocrinology and psychology departments, responsible for disseminating health knowledge and addressing patient inquiries. The working team comprised senior nurses, specialized endocrinology nurses, and online platform administrators, primarily overseeing the management and daily operations of the platform.

(3) Specific Service Contents: All the participants in the observation group were actively encouraged to use the platform. On the day of discharge, patients were invited and guided to follow the WeChat official account, instructed on how to navigate various sections, assisted in setting up personal profiles, and trained in the use of blood glucose monitors with a great desire to participate in the study. Patients were also advised to promptly send their blood glucose readings to the nurse via WeChat, enabling the nursing staff to update their personal profiles in a timely manner. The participants contacted healthcare professionals on a weekly basis and a senior nurse was responsible for updating the patient’s disease status weekly, allowing both patients and their families to stay informed about the progress of extended care services in real time. The guidance team developed and updated health education materials in the form of images, text, and videos, which were promptly uploaded to the health column by the working team. The interactive communication section provided a platform for patients to engage with one another, encouraging active participation and positive interactions with extended care services. The real-time online guidance section facilitated scheduled video consultations each week, offering professional psychological counseling and medical advice. Additionally, the online follow-up appointment scheduling feature was designed to allow patients to conveniently book follow-up visits based on their personal schedules.

### Observation indicators

(1) The blood glucose levels of both groups were compared at discharge and three months after the intervention. Specifically, fasting blood glucose (FBG), 2-hour postprandial blood glucose (2hPG), and glycated hemoglobin (HbA1c) levels were measured by collecting fasting venous blood samples in the early morning and 2-hour postprandial venous blood samples.

(2) The emotional state of both groups was assessed at discharge and three months after the intervention using the Self-Rating Anxiety Scale (SAS) [9] and the Self-Rating Depression Scale (SDS) [10]. Both SAS and SDS consist of 20 items, each rated on a four-point Likert scale from 1 to 4. An SAS score greater than 50 indicates the presence of anxiety, while an SDS score greater than 53 indicates the presence of depression. Higher scores on both scales indicate more severe levels of anxiety or depression in patients.

(3) The self-management ability of both groups was assessed at discharge and three months after the intervention using the Summary of Diabetes Self-Care Activities (SDSCA) [11]. The SDSCA evaluates patients across five dimensions: blood glucose monitoring, diet control, exercise, foot care, and medication adherence, with a total of 11 items. Each item is rated on a scale from 1 to 7, with higher scores indicating greater self-management ability.

(4) The quality of life of both groups was assessed at discharge and three months after the intervention using the General Quality of Life Inventory-74 (GQOL-74) [12]. The GQOL-74 primarily evaluates four dimensions: physical function, psychological function, social function, and material living status. Each dimension is scored out of 100 points, with higher scores reflecting better quality of life.

(5) The incidence rates of hypoglycemia and readmission were compared between the two groups within three months after discharge by recording the occurrences of hypoglycemia and subsequent hospitalizations during this period.

### Statistical methods

The statistical software SPSS 26.0 was used for data processing and analysis. Categorical data were presented as rates, with differences compared using the chi-squared test or Fisher’s exact probability test. Continuous data were expressed as mean ± standard deviation (x̅ ± s), with differences analyzed using the t-test. A p-value < 0.05 was considered statistically significant.

## Results

### Blood glucose level comparison

Three months after the intervention, FBG, 2hPG, and HbA1c levels in both groups decreased compared to discharge levels, with the observation group exhibiting significantly lower values (*p* < 0.05). Details are presented in Table 2.

**Table 2:** Comparison of blood glucose levels between the two groups at discharge and 3 months post-intervention

Group	Number of cases	FBG		2hPG			HbA1c		
		At discharge	3 months post-intervention	At discharge	3 months post-intervention		At discharge		3 months post-intervention
Observation group	75	8.11 ± 2.08	5.78 ± 0.65	10.89 ± 1.57	8.56 ± 1.39	8.25 ± 1.62		6.88 ± 0.81	
Control group	75	8.18 ± 2.05	6.03 ± 0.71	11.02 ± 1.42	9.07 ± 1.57	8.31 ± 1.59		7.17 ± 0.87	
*t*		0.208	2.249	0.532	2.106	0.229		2.113	
*P*		0.836	0.026	0.596	0.037	0.819		0.036	

### Emotional state comparison

Three months after the intervention, SAS and SDS scores in both groups declined compared to discharge levels, with the observation group exhibiting significantly lower scores (*p* < 0.05). Details are presented in Table 3.

**Table 3:** Comparison of emotional state between the two groups at discharge and 3 months post-intervention

Group	Number of cases				
		At discharge	3 months post-intervention	At discharge	3 months post-intervention
Observation group	75	51.48 ± 4.26	41.57 ± 3.82	53.95 ± 5.12	35.03 ± 4.47
Control group	75	52.33 ± 5.09	43.06 ± 4.13	54.18 ± 4.63	38.31 ± 4.75
*t*		1.109	2.294	0.289	4.355
*P*		0.269	0.023	0.773	0.000

### Self-management ability comparison

Three months after the intervention, SDSCA scores across all dimensions increased in both groups compared to discharge levels, with the observation group demonstrating significantly higher scores in all dimensions (*p* < 0.05). Details are presented in Table 4.

**Table 4:** Comparison of Self-management ability between the two groups at discharge and 3 months post-intervention

Group	Number of cases	Blood glucose monitoring		Dietary control		Exercise	
		At discharge	3 months post-intervention	At discharge	3 months post-intervention	At discharge	3 months post-intervention
Observation group	75	7.33 ± 1.06	5.39 ± 1.23	7.15 ± 1.29	5.31 ± 1.19	6.48 ± 1.42	5.03 ± 1.18
Control group	75	7.25 ± 1.11	4.48 ± 1.18	7.07 ± 1.32	4.63 ± 1.22	6.53 ± 1.36	4.15 ± 1.06
*t*		0.451	4.624	0.375	3.455	0.220	4.805
*P*		0.652	0.000	0.708	0.001	0.826	0.000

### Quality of life comparison

Three months after the intervention, GQOL-74 scores across all dimensions increased in both groups compared to discharge levels, with the observation group demonstrating significantly higher scores in all dimensions (*p* < 0.05). Details are presented in Table 5.

**Table 4a:** Continued from Table 4: comparison of Self-management ability between the two groups at discharge and 3 months post-intervention

Group	Number of cases	Foot care		Medication adherence	
		At discharge	3 months post-intervention	At discharge	3 months post-intervention
Observation group	75	6.67 ± 1.43	4.69 ± 1.21	6.08 ± 1.16	4.31 ± 1.05
Control group	75	6.55 ± 1.36	4.02 ± 1.08	6.23 ± 1.28	3.87 ± 1.18
*t*		0.527	3.578	0.752	2.412
*P*		0.599	0.001	0.453	0.017

### Hypoglycemia and readmission rate comparison

The incidence rates of hypoglycemia and readmission were lower in the observation group than in the control group (*p* < 0.05). Details are presented in Table 6.

**Table 5:** Comparison of quality of life between the two groups at discharge and 3 months post-intervention

Group	Number of cases	Physical function		Psychological function		Social function		Material living status	
		At discharge	3 months post-intervention	At discharge	3 months post-intervention	At discharge	3 months post-intervention	At discharge	3 months post-intervention
Observation group	75	58.32 ± 4.37	62.88 ± 4.19	56.59 ± 5.03	61.28 ± 4.19	58.06 ± 4.35	62.76 ± 4.65	60.31 ± 4.52	63.97 ± 4.36
Control group	75	59.61 ± 5.03	65.73 ± 4.26	55.87 ± 5.12	63.12 ± 5.33	57.81 ± 5.19	66.13 ± 4.37	60.59 ± 4.62	67.23 ± 4.81
*t*		1.677	4.131	0.869	2.350	0.320	4.574	0.375	4.349
*P*		0.096	0.000	0.386	0.020	0.750	0.000	0.708	0.000

**Table 6:** Comparison of hypoglycemia and readmission between the two groups [n (%)]

Group	Number of cases	Hypoglycemia	Readmission
Observation group	75	1	0
Control group	75	7	6
Fisher’s exact test		-	-
*p*-value		0.034	0.028

Hypoglycemia was defined as blood glucose < 2.8 mmol/L

## Discussion

With advancements in technology and evolving societal trends, patients’ expectations for healthcare services have increased, driving ongoing improvements in medical and nursing systems. Extended care has demonstrated benefits in managing chronic diseases [13]. Diabetes, as a prevalent chronic disease, is linked to factors such as unhealthy dietary and exercise habits and insufficient knowledge of disease prevention and management [14]. In recent years, the expansion of internet technology has facilitated the widespread integration of online platforms like WeChat into healthcare. As a result, internet-based extended care services have gradually emerged as an innovative nursing model. This study primarily evaluates the effectiveness of a diabetes chronic disease management platform in delivering extended care for patients with diabetes.

Previous studies have indicated that extended care can improve blood glucose control in patients [15]. The findings of this study indicate that three months after the intervention, FBG, 2hPG, and HbA1c levels were lower in both groups compared to discharge, with significantly lower levels observed in the intervention group. These results indicate that the diabetes chronic disease management platform enhances the effectiveness of extended care in improving blood glucose control.

Traditional extended care services primarily rely on telephone follow-ups, which are associated with high refusal rates and missed visits. Additionally, these follow-ups are largely nurse-led, with minimal physician involvement, making it challenging to achieve optimal follow-up outcomes and effectively regulate blood glucose levels [16]. In contrast, the diabetes chronic disease management platform utilizes the WeChat official account to deliver diabetes-related education through text, images, and videos across multiple sections. This approach accommodates patients’ schedules by overcoming time, location, and financial constraints. Moreover, it enables healthcare professionals to monitor patients’ blood glucose test results in real time, provide timely interpretation, and assist in adjusting their dietary and exercise plans, thereby enhancing blood glucose management [17]. 

Furthermore, three months after the intervention, SAS and SDS scores in both groups declined when compared to discharge, with the intervention group showing a significantly greater reduction. These findings indicate that the diabetes chronic disease management platform also enhances the emotional well-being of patients with diabetes. Chronic hyperglycemia can lead to vascular damage and increase the risk of complications such as cardiovascular and kidney diseases, particularly in middle-aged and older adults. These complications place significant physical, psychological, and financial burdens on patients and their families, making them more susceptible to anxiety and depression [18, 19]. 

If these psychological issues are not promptly addressed, they may worsen over time, negatively impacting health outcomes, reducing treatment adherence, and further impairing blood glucose control, ultimately creating a cycle of worsening psychological and metabolic health [20]. Traditional extended care relies primarily on nurses and lacks professional psychological support [20]. In contrast, the WeChat-based diabetes management platform incorporates a multidisciplinary team of experts, including specialists in psychology and endocrinology, who collaborate to provide personalized guidance and psychological counseling. This multidisciplinary approach helps alleviate emotional distress, improve disease management, and enhance patient confidence in treatment, leading to significant improvements in emotional well-being [21]. 

Three months after the intervention, the SDSCA scores in all dimensions for both groups increased compared to those at discharge, with the observation group exhibiting significantly greater improvements in all aspects (*p* < 0.05). These results indicate that the diabetes chronic disease management platform further strengthens the impact of extended care on enhancing self-management ability and quality of life for patients with diabetes.

The WeChat-based diabetes management platform offers structured guidance in blood glucose monitoring, dietary regulation, physical activity, follow-up scheduling, and peer interaction. Disease-related knowledge is provided in various formats, including text and images, making it more accessible and comprehensible. This approach promotes patient engagement while enabling better retention of diabetes management knowledge. Additionally, the educational content can be saved and revisited, reinforcing learning over time [22]. Moreover, real-time interaction between healthcare providers and patients is facilitated through the platform, allowing prompt guidance and supervision based on individual patient conditions. This interactive model helps establish effective self-management practices, ultimately enhancing patient autonomy in disease control.

Strengthening self-management not only supports better blood glucose regulation but also positively influences psychological well-being, disease awareness, and overall health outcomes. As a result, it contributes to better long-term disease control, reducing complications that affect other organ systems and minimizing the impact on patients’ quality of life. Additionally, the study findings indicate that the incidence of hypoglycemia and hospital readmission was lower in the observation group than in the control group (*p* < 0.05). These results confirm that extended care via the WeChat platform supports the adoption of scientifically guided dietary and lifestyle habits, leading to more stable blood glucose levels, improved disease control, and a reduced risk of hypoglycemia and readmission.

However, the current application and promotion of the WeChat diabetes management platform in clinical settings still have certain limitations. The implementation of such a care model requires the presence of relevant professionals to provide timely guidance and communication to patients, and patients also need to be proficient in using smart software such as WeChat. Therefore, it requires a certain amount of human and material resources, and there are certain requirements for patients. As a result, there are certain application limitations for hospitals with limited human resources or patients who have difficulty using digital platforms.

In summary, integrating extended care services for patients with diabetes through the WeChat-based diabetes management platform proves to be a more effective strategy for enhancing disease understanding and management. This approach supports patients in modifying their diet, exercise routines, and other lifestyle behaviors while ensuring adherence to prescribed medications and blood glucose monitoring. As a result, it facilitates better blood glucose regulation, improves psychological well-being, strengthens self-management abilities, and reduces the risk of hypoglycemia and hospital readmission.

## Data Availability

All data generated or analysed during this study are included in this article. Further enquiries can be directed to the corresponding author.

## Abbreviations

FBG: Fasting blood glucose
2hPG: 2-hour Postprandial blood glucose
HbA1c: Glycated hemoglobin
SAS: Self-Rating Anxiety Scale
SDS: Self-Rating Depression Scale
SDSCA: Summary of Diabetes Self-Care Activities
GQOL-74: Generic Quality of Life Inventory-74
